# Fluoroless coronary sinus cannulation using a BeeAT catheter and the CARTO mapping system

**DOI:** 10.1016/j.ipej.2025.08.007

**Published:** 2025-08-20

**Authors:** Shintaro Yamagami, Tsukasa Motoyoshi, Takashi Kanda, Toshihiro Tamura

**Affiliations:** a Department of Cardiology, Tenri Hospital, Tenri, Nara, Japan; b Cardiovascular Division, Advanced Cardiac Rhythm Management Center, Osaka Keisatsu Hospital, Osaka, Japan

**Keywords:** Fluoroless ablation, Coronary sinus cannulation, CARTO mapping, Intracardiac echocardiography, BeeAT catheter, Femoral approach

## Abstract

Fluoroscopy-free ablation techniques have gained popularity in recent years. However, coronary sinus (CS) cannulation via the femoral approach remains technically challenging, particularly when using specialized catheters like the BeeAT™. To demonstrate a reproducible technique for fluoroscopy-free CS cannulation using the BeeAT catheter guided by CARTO® electroanatomical mapping and intracardiac echocardiography (ICE). Forty three patients undergoing atrial fibrillation ablation were enrolled. After 3D mapping of the right atrium and CS ostium identification via ICE, a femoral-type BeeAT catheter was inserted. Direct advancement or RA loop techniques were applied based on anatomy. CARTO settings were adjusted to allow impedance-based catheter visualization. Successful CS cannulation without fluoroscopy was achieved in all 43 cases. The direct technique succeeded in 36 cases, and the RA loop method was used in 7. No complications occurred.

**Conclusion:**

Fluoroscopy-free femoral CS cannulation with the BeeAT catheter is safe, feasible, and enhances procedural efficiency while avoiding radiation exposure.

Radiation exposure is a significant concern in catheter-based electrophysiological procedures. In recent years, fluoroscopy-free catheter ablation techniques have been increasingly adopted for various arrhythmias, particularly atrial fibrillation (AF). This shift has been accompanied by a broader application of non-fluoroscopic insertion of diagnostic catheters, including coronary sinus (CS) catheters required for ablation procedures [1,2].

The BeeAT™ catheter (Japan Lifeline, Tokyo, Japan) is a steerable 20-pole diagnostic catheter designed to simultaneously record electrical activity from both the right atrium (RA) and the CS, with its proximal 10 electrodes positioned in the RA and distal 10 electrodes in the CS (Fig. 1-A). This dual-functionality allows procedures to be performed with fewer catheters, thereby contributing to a simpler and less invasive workflow.

**Fig. 1:**
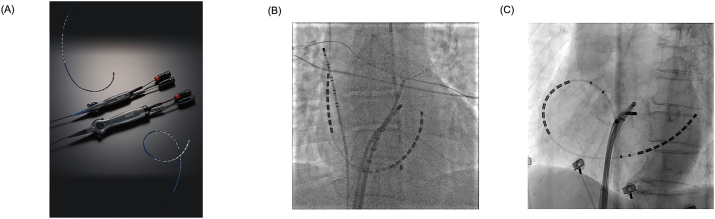
**Comparison of CS cannulation routes and the external design of the BeeAT™ catheter.** A: External view of the BeeAT™ catheter, a 20-pole steerable diagnostic catheter designed for simultaneous recording of right atrial and CS signals. B: Fluoroscopic image showing CS cannulation using the BeeAT™ catheter via the right internal jugular vein approach, favoring alignment with the CS axis. C: Fluoroscopic image showing CS cannulation using the femoral vein approach. Although this approach is more comfortable for patients, it can be technically challenging in the absence of fluoroscopy.

Two commercially available types of BeeAT™ catheters exist: one designed for right internal jugular vein access (Fig. 1-B) and another suitable for femoral vein access (Fig. 1-C). The internal jugular approach offers easier catheter alignment with the CS but may cause patient discomfort and carries risks, including pneumothorax [3]. Conversely, femoral access offers significant patient comfort and mitigates complications associated with internal jugular vein access. However, it presents technical challenges owing to less direct anatomical alignment with the CS. As shown in Fig. 1-C, femoral access often requires forming a loop configuration—similar to an α-loop—within the RA to engage the CS ostium. This maneuver is particularly challenging in fluoroscopy-free settings and may inevitably increase fluoroscopy time. Therefore, we propose a fluoroscopy-free technique for femoral CS cannulation using the BeeAT™ catheter, guided by the CARTO® 3 electroanatomical mapping system (Biosense Webster, Irvine, CA, USA).

This technique was performed on 43 consecutive patients who underwent catheter ablation for AF. After femoral venous access, a three-dimensional electroanatomical map of the RA, inferior vena cava, superior vena cava, and CS was first created using the CARTO® 3 system (Biosense Webster, Irvine, CA, USA) and a PENTARAY™ catheter (Biosense Webster) (Fig. 2). The CS ostium was localized using a SOUNDSTAR™ intracardiac echocardiography (ICE) catheter (Biosense Webster), and its position was marked on the electroanatomical map (Fig. 3). After creating the electroanatomical map of the right heart structures, we used the SOUNDSTAR™ catheter to visualize the cavotricuspid isthmus and the CS ostium. These two anatomical landmarks were then roughly aligned with the pre-acquired CT image for integration. Subsequently, a steerable 20-pole BeeAT™ catheter was inserted and guided toward the marked ostium of the CS based on the completed matrix. Although the BeeAT™ catheter typically does not appear on the three-dimensional (3D) map unless all 20 electrodes are within the anatomical matrix, we modified the CARTO display settings such that each electrode could be visualized based on its individual impedance data. As a result, the BeeAT™ catheter remained visible on the 3D map even when not all 20 electrodes were fully encompassed within the anatomical shell, appearing as a continuous “string-of-beads” configuration (Fig. 4A). This is the default rendering method when the anatomical matrix is limited. However, when all 20 electrodes were encompassed within a sufficiently detailed anatomical matrix, the catheter was rendered as a smooth, continuous structure, more closely resembling its actual shape, and enhancing the accuracy of real-time navigation (Fig. 4B). During the placement of the BeeAT™ catheter in the RA and CS, one of the above display modes was consistently used to ensure continuous visualization of the catheter on the 3D map. Additionally, the 3D map was fixed at two standard fluoroscopic projection angles, the right and left anterior oblique views, to replicate conventional fluoroscopy-guided manipulation.

**Fig. 2:**
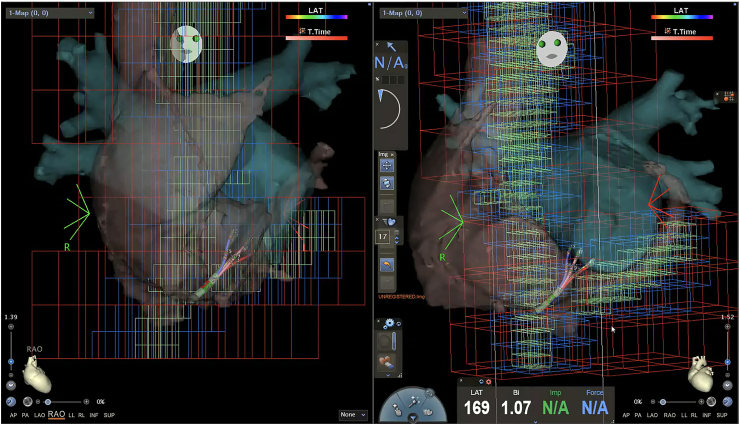
**Three-dimensional electroanatomical mapping of the RA using a PENTARA catheter.** The anatomical matrix of the inferior vena cava, RA, superior vena cava, and CS was created prior to catheter insertion. This map aids in orientation and navigation during a fluoroscopy-free approach.

**Fig. 3:**
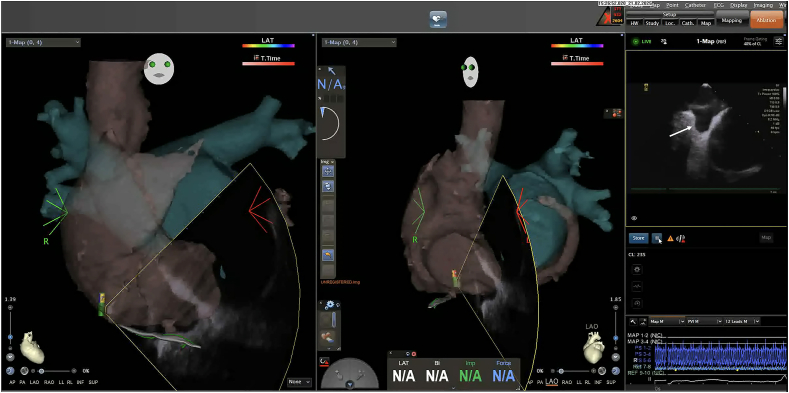
**Three-dimensional electroanatomical mapping of the RA using a SOUNDSTAR catheter.** The coronary sinus (CS) was clearly visualized and annotated on a map to guide catheter placement.

**Fig. 4:**
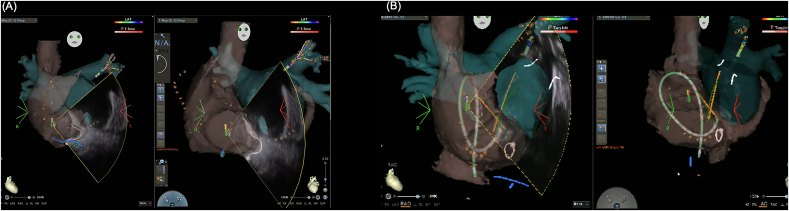
**Visualization of the BeeAT™ catheter on CARTO ® 3 system.** A: Normally, the BeeAT™ catheter appears as a “string-of-beads,” representing its 20 electrodes. B: When all 20 electrodes of the BeeAT™ catheter were within an area with sufficient anatomical matrix data, the catheter appeared as a smooth, continuous structure, closely resembling its actual form and enabling improved real-time navigation.

With respect to the placement of the BeeAT™ catheter, two different techniques were employed depending on the anatomical orientation. In all cases, a direct advancement technique was initially attempted by guiding the catheter tip directly into the CS. The BeeAT™ catheter was then looped within the RA to achieve stable positioning, allowing for simultaneous recording of both the RA and CS. If this standard approach proved difficult, an alternative method involving the formation of an α-loop within the RA to facilitate CS entry (the RA loop technique) was used. During real-time ICE monitoring, we observed the catheter tip approaching and entering the CS (as illustrated in the accompanying Video 1). A stable loop configuration was achieved without the need for fluoroscopy, resulting in successful catheter positioning in all 43 patients without any complications. Among the 43 cases, the standard approach was successful in 36 patients, whereas the RA loop technique was required in the remaining 7 cases. Identification of the CS ostium using ICE was straightforward and could be completed within 30 seconds in all cases. The time required for BeeAT™ catheter insertion was 55 ± 9 seconds on average for the 36 cases where the standard approach was successful, and 113 ± 18 seconds for the 7 cases where the RA loop method was used after an unsuccessful attempt with the standard approach. The video also demonstrates both techniques, highlighting their feasibility under completely fluoroscopy-free conditions.

The femoral-type BeeAT™ catheter offers the distinct advantage of enabling simultaneous recording of the right atrial and CS signals using a single catheter. However, compared with conventional CS catheters, their structural complexity, such as length and multipolar design, makes accurate positioning more technically demanding, especially from the femoral approach. Consequently, fluoroscopic guidance is essential for its placement.

In this case series, we demonstrated that fluoroscopy-free CS cannulation with a BeeAT™ catheter using the femoral approach is not only feasible but also reproducible when guided by electroanatomical mapping and ICE. By enabling consistent 3D visualization and real-time anatomical feedback, this method provides a high level of procedural control without radiation. Regarding the learning curve, we believe it depends on the operator's prior experience with inserting the BeeAT™ catheter via the IVC under fluoroscopy. Since the catheter can be visualized similarly on 3D mapping as under fluoroscopy, operators with such experience would face minimal learning curve. In fact, because the CS ostium is clearly visualized on the 3D map, unlike with fluoroscopy, we believe it may even facilitate faster cannulation in certain cases where the ostium is difficult to identify fluoroscopically.

Notably, all 43 procedures were successfully completed without complications, thereby supporting its safety profile.

Integrating this fluoroscopy-free technique into routine clinical practice can reduce the reliance on fluoroscopy, streamline procedural workflows by minimizing catheter exchanges, and enhances radiation safety for both patients and staff. This approach is particularly beneficial for centers aiming for zero or near-zero fluoroscopy ablation strategies, as it mitigates radiation exposure risks and reduces the need for lead aprons, thereby minimizing associated orthopedic complications among medical personnel. Although the BeeAT™ catheter is moderately more expensive than standard decapolar CS catheters, its ability to record both RA and CS signals simultaneously, along with its capacity for internal cardioversion, may justify its use in selected cases. These include complex ablation procedures or patients in whom minimizing venous access is desirable. In Japan, the catheter is widely used in AF ablation procedures, and its integration into a fluoroscopy-free workflow can contribute to both procedural efficiency and safety.

## Patient consent statement

Individual patient consent was waived, as no identifiable information is included and institutional approval for publication was granted.

## Ethical statement

This study was conducted in accordance with the principles outlined in the Declaration of Helsinki.

Ethical approval was waived as this was a retrospective observational study without any identifiable patient information and with no intervention beyond standard clinical care.

Institutional approval for data analysis and publication was obtained at Tenri Hospital.

## Funding

None.

## Declaration of competing interest

All authors declare no conflicts of interest.
